# Nitrogen-Doped Graphene with Enhanced Room Temperature NH_3_ Gas Sensing Properties

**DOI:** 10.3390/nano16161044

**Published:** 2026-08-21

**Authors:** Qingwu Huang, Peng Zhou, Wulin Song, Jinjin Wu

**Affiliations:** 1 Analytical and Testing Center, Huazhong University of Science and Technology, No. 1037, Luoyu Road, Wuhan 430074, China; qwhuang@hust.edu.cn (Q.H.); zhoupeng_peng@hust.edu.cn (P.Z.); wulins@126.com (W.S.); 2 State Key Laboratory of Materials Processing and Die & Mould Technology, Huazhong University of Science and Technology, No. 1037, Luoyu Road, Wuhan 430074, China

**Keywords:** room-temperature sensor, NH_3_ gas sensing performance, pyridinic N, graphitic N, DFT calculations, synergistic effect

## Abstract

Herein, nitrogen-doped wrinkled multilayer graphene sheets (N-doped graphene) are synthesized via a facile solvothermal route followed by NH_3_ atmosphere annealing at different temperatures. Systematic characterization by SEM, XRD, FTIR, Raman, and XPS confirms the successful incorporation of nitrogen with tunable configurations, i.e., pyridinic N, pyrrolic N, and graphitic N, whose relative fractions are strongly dependent on annealing temperature. Room-temperature gas sensing tests toward NH_3_ reveal that the G-600 (N-doped graphene annealed at 600 °C) sensor exhibits the highest response (4.76 toward 300 ppm NH_3_), about 7.8 times that of G-400 (a counterpart sample thermally treated at 400 °C), along with excellent selectivity, reproducibility, and stability. Density functional theory (DFT) calculations demonstrate that pyridinic N provides the strongest adsorption affinity for NH_3_ and the largest charge transfer, whereas graphitic N ensures superior electrical conductivity. The optimal performance of G-600 arises from a synergistic effect between abundant pyridinic N active sites for enhanced NH_3_ adsorption and sufficient graphitic N for efficient charge transport. This work not only elucidates the mechanism of nitrogen-configuration-governed sensing behavior but also offers a rational strategy for designing high-performance room-temperature NH_3_ sensors based on N-doped graphene.

## 1. Introduction

Ammonia (NH_3_) is widely used as an important chemical raw material and industrial refrigerant in agriculture, the chemical industry, and pharmaceuticals [1]. Meanwhile, NH_3_ has a hydrogen storage capacity of up to 17.7 wt%, making it a promising hydrogen carrier and an effective means for large-scale, long-distance hydrogen storage and transportation [2]. However, NH_3_ is toxic, flammable, and explosive; both long-term and short-term exposure can cause serious harm to human health. The American Conference of Governmental Industrial Hygienists (ACGIH) has set a long-term exposure limit of 25 parts per million (ppm) and a short-term exposure limit of 35 ppm [3], but the human olfactory threshold is approximately 53 ppm [4], implying that hazardous concentrations may not be detected in time. Therefore, developing high-performance NH_3_ sensing technology for real-time and accurate monitoring is of great significance for industrial safety, environmental monitoring, and the safe operation of the hydrogen energy industry chain.

Although conventional metal oxide semiconductor (MOS) gas sensors have been extensively studied, they typically require high operating temperatures in the range of 200–400 °C to achieve sufficient sensitivity and response kinetics [3]. Their high operating temperature not only introduces high energy consumption and thermal management issues but also increases safety risks in flammable and explosive environments, limiting their application in portable, wearable, and large-scale distributed monitoring scenarios [3,5]. In contrast, sensors that can operate at room temperature do not require external heating elements, which significantly reduces power consumption, improves system safety, and simplifies device architecture, thereby facilitating integration into miniaturized, low-cost detection equipment to meet the growing demand for real-time on-line monitoring [5,6]. Consequently, the development of room-temperature NH_3_ sensors with both high sensitivity and good selectivity has become a research hotspot in this field.

In recent years, graphene has been regarded as a highly promising gas sensing material due to its unique two-dimensional honeycomb structure, extremely high specific surface area (theoretical value ~2630 m^2^/g), and excellent electrical conductivity [7,8]. However, pristine graphene exhibits only weak physical adsorption toward most gas molecules and lacks effective chemical active sites, resulting in low gas sensing response and poor selectivity, which severely limit its practical applications [1,5]. To overcome this limitation, various modification strategies have been explored, including algorithm-based signal compensation research, chemical doping, functionalization, compositing, and the construction of chemical bonding interfaces [8,9,10]. For example, by compositing graphene with semiconductors such as ZnO quantum dots, WO_3_, or TiO_2_, and utilizing photoexcitation or chemical bonding (e.g., C–Ti, C–Bi, and C–W bonds), its room-temperature gas sensing performance toward volatile organic compounds such as formaldehyde and toluene can be significantly enhanced [11,12,13,14]. Among these approaches, nitrogen doping is widely recognized as an effective means to tune the electronic structure and chemical properties of graphene, because nitrogen atoms have a similar size to carbon atoms and can be incorporated into the graphene lattice in three representative configurations, i.e., edge-located pyridinic N, five-membered-ring pyrrolic N, and basal-plane substituted graphitic N [15,16]. Theoretical studies have shown that nitrogen doping can introduce band gaps, generate structural defects, and enhance conductivity in graphene, all of which are beneficial for improving gas sensing performance [17].

Although previous studies have verified the promising potential of N-doped graphene for room-temperature NH_3_ sensing [17], the manner in which different nitrogen doping configurations (i.e., graphitic N, pyridinic N, and pyrrolic N) regulate room-temperature gas sensing performance, as well as the underlying mechanisms governing NH_3_ adsorption and sensing response, has yet to be systematically explored. Against this research backdrop, nitrogen-doped wrinkled multilayer graphene sheets based on NH_3_ sensors were fabricated in this work, and their surface morphology, microstructure, and chemical composition were systematically characterized. The effects of diverse nitrogen doping configurations on the room temperature NH_3_ sensing behaviors were comprehensively investigated. Furthermore, DFT calculations were performed to analyze the adsorption energy and structural parameters of the N-doped graphene NH_3_ system. Atomic-level insights were acquired to clarify the intrinsic mechanisms of nitrogen doping configurations modulating NH_3_ adsorption and room-temperature sensing performance. The findings of this study are expected to provide credible theoretical guidance for the optimization of nitrogen doping configurations and the performance regulation of high efficiency room-temperature NH_3_ sensors based on N-doped graphene. This strategy also offers an efficient route to fabricate gram-scale N-doped graphene nanosheets, enabling the achievement of favorable functional properties unattainable for pristine graphene.

## 2. Materials and Methods

### 2.1. Synthesis of Samples

Following our previous reports [5,11], the typical experimental procedure is described as follows. Sodium metal (3 g, >99.7 wt% purity, Aladdin, Shanghai, China) and glucose (3 g, Aladdin, Shanghai, China) were dissolved in absolute ethanol (30 mL, Sinopharm, Beijing, China) and then transferred into a Teflon-lined stainless-steel autoclave (Model 4843, Parr Instrument Co., Champaign, IL, USA). The mixture was heated at 220 °C for 72 h in the sealed reactor. The resulting gray precursor was collected and subsequently pyrolyzed rapidly at 300 °C in a muffle furnace (Model YH-1200, Wuhan Yahua Electric Furnace Co., Ltd., Wuhan, China) under an NH_3_ atmosphere. The NH_3_ annealing treatment was then carried out in a tube furnace (Model YH-G1200, Wuhan Yahua Electric Furnace Co., Ltd., Wuhan, China). Specifically, the as-prepared precursor was placed in a quartz boat (Wuhan Dashan Precision Furnace Industry Co., Ltd., Wuhan, China) positioned at the center of the tube furnace. After purging with NH_3_ for about 10 min, the furnace was heated to 150 °C within 10 min, and then directly raised to the target annealing temperature. The temperature was maintained at the desired value for 50 min, after which the furnace was allowed to cool to room temperature under a continuous NH_3_ flow. Three different reaction temperatures were employed: 400, 600, and 800 °C. The resulting black powders were thoroughly washed with deionized water and ethanol several times, filtered, and finally dried under vacuum. For clarity, the products obtained at 400, 600, and 800 °C were denoted as G-400, G-600, and G-800, respectively.

### 2.2. Materials Characterization

The crystalline structure and phase purity of the samples were examined by X-ray diffraction (XRD) on a Philips X’Pert Pro diffractometer with Cu Kα radiation (λ = 0.15416 nm) operating at 40 kV and 30 mA. Raman spectra were recorded on a Horiba Jobin Yvon LabRAM HR800 Raman spectrometer (Horiba Jobin Yvon, Paris, France) equipped with a charge-coupled device (CCD) detector and a 300 mW green laser (532 nm). Fourier-transform infrared (FT-IR) spectra were obtained using a PerkinElmer FT-IR spectrometer. The morphology was observed by high-resolution field-emission scanning electron microscopy (FE-SEM, FEI Sirion 200, FEI Company, Hillsboro, OR, USA) with an Oxford Instruments X-Max energy-dispersive X-ray detector attached to the SEM. Surface chemical states were analyzed by X-ray photoelectron spectroscopy (XPS) on a Kratos Axis Ultra DLD spectrometer (Kratos Company, Manchester, UK) using monochromatic Al Kα radiation.

### 2.3. Gas Sensor Fabrication and Testing

The gas sensing measurements were conducted following a procedure partially described in our previous work [6,18]. Alumina flat substrates (6 mm × 8 mm) with pre-printed Au interdigital electrodes were used as sensor platforms. A methanol suspension of the as-prepared sample was gently dropped onto the substrate using a dropper and then dried for 5 h to evaporate the solvent. To improve the contact between the film and the electrodes, the device was vacuum-annealed at 180 °C for 2 h. Subsequently, the Au electrodes were connected to the Au pedestal via a gold wire bonder. The sensor was placed in a stainless-steel chamber, where a mixture of NH_3_ and dry air flowed over the device at atmospheric pressure and room temperature, with flow rates controlled by mass flow controllers. The conductance variation was recorded using a. computer-controlled data acquisition card (Model 6008, National Instruments Corp., Austin, TX, USA) The gas sensing response was defined as the relative conductance change, S = |Gg − Ga|/Ga, where Gg and Ga denote the electrical conductance of the sensor in the target gas and in dry air, respectively.

### 2.4. DFT Calculation

DFT calculations were performed using the Gaussian 09W software (Revision D.01, Gaussian, Inc., Wallingford, CT, USA) package to investigate the electron transfer behavior of N-doped graphene during gas sensing detection. All geometric optimizations were carried out at the DFT/B3LYP-D3(BJ) level with the 6–31G basis set, where the D3(BJ) empirical van der Waals dispersion correction was included to accurately capture long-range intermolecular dispersion interactions between NH_3_ and N-doped graphene, following the protocols reported in previous literature [19]. To clarify the intermolecular interactions between NH_3_ and N-doped graphene with different nitrogen configurations (graphitic N, pyridinic N, and pyrrolic N) at 298.15 K, adsorption models were constructed separately for each configuration. The calculation workflow was arranged as follows. Firstly, the isolated N-doped graphene model and the NH_3_ molecule were geometrically relaxed independently with dispersion correction activated. Subsequently, composite systems consisting of the fully relaxed N-doped graphene and NH_3_ were built and re-optimized under identical computational parameters. After convergence, the counterpoise method was applied to correct the basis-set superposition error (BSSE). All adsorption energies reported in this work are BSSE-corrected values. Structural parameters, molecular orbital information, bond lengths, total optimized energies, and Mulliken charges were extracted for further mechanistic analysis.

### 2.5. Electrical Characterization

Electrical properties were characterized using an Ecopia HMS-5300 Hall measurement system under ambient conditions (300 K, atmospheric pressure). The measurements were performed on N-doped samples deposited on quartz substrates utilizing the standard van der Pauw four-probe method. The as-synthesized powder was fully ground and cold-pressed into compact square thin wafers (10 × 10 mm) under 10 MPa pressure, followed by vacuum annealing at 150 °C to eliminate internal microcracks. The film thickness was precisely measured using a digital micrometer with 1 μm resolution under repeated measurements for Hall coefficient calculation. Ohmic contacts were established by pressing indium metal dots onto the four corners of the samples. To minimize experimental errors, the system applied alternating positive and negative magnetic fields to eliminate offset voltage errors and automatically calculated the sheet resistance. The Hall mobility and carrier type were automatically extracted. The final reported values represent the average of three repeated measurements.

## 3. Results and Discussion

### 3.1. Characterization of N-Doped Graphene

**Figure 1:**
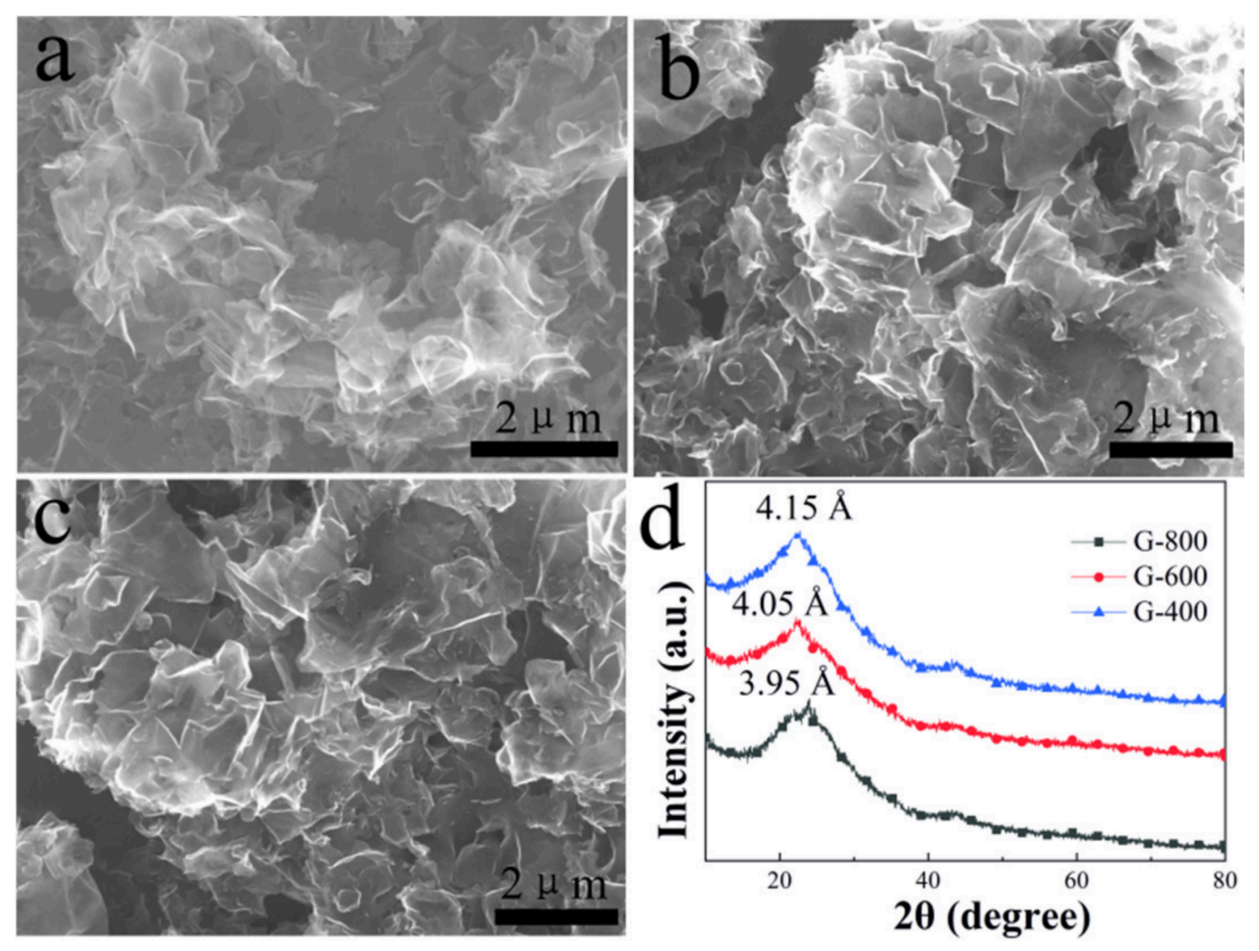
Typical SEM images of N-doped graphene samples prepared at different temperatures ((**a**) G-400, (**b**) G-600, (**c**) G-800) and the corresponding XRD patterns (**d**).

Typical FESEM images of the G-400, G-600, and G-800 samples are shown in Figure 1a–c. As previously reported [5], the as-prepared N-doped graphene samples are silk-like. As shown in Figure 1a–c, they have micrometer-long wrinkles with overlap at the edges and demonstrate nanosheets on their surface.

The XRD patterns of all samples are presented in Figure 1d. The (002) diffraction peaks of the three samples all exhibit obvious broadening, which reveals low structural ordering along the layer stacking direction. Meanwhile, each sample displays a broad yet distinguishable diffraction peak at approximately 23° (2θ), which can be assigned to the (002) crystal plane. The average crystallite stacking thickness Lc is calculated via the Scherrer equation: D = Kλ/βcosθ, where K denotes the shape factor (set as 0.90 for amorphous carbon), λ represents the wavelength of X-ray, θ is the Bragg diffraction angle, and β stands for the full width at half maximum (FWHM) of the diffraction peak in radians.

The interlayer spacing d{002} values of the G-400, G-600, and G-800 samples are 4.15 Å, 4.05 Å, and 3.95 Å, respectively. In contrast, the d{002} of pristine graphene is only 3.4 Å, which is smaller than that of the as-prepared samples. Oxygen-containing and/or nitrogen-containing functional groups grafted on both sides of graphene sheets can enlarge the interlayer distance; thus, all samples fabricated in this work possess larger interlayer spacing than pristine graphene. The increased d{002} values suggest that organic radicals are anchored on the surface of samples with different degrees [20]. Among them, the G-400 sample with the maximum d{002} has the highest grafting density of radicals. Similar variation trends of d{002} have also been reported in the previous literature [21,22,23,24].

**Figure 2:**
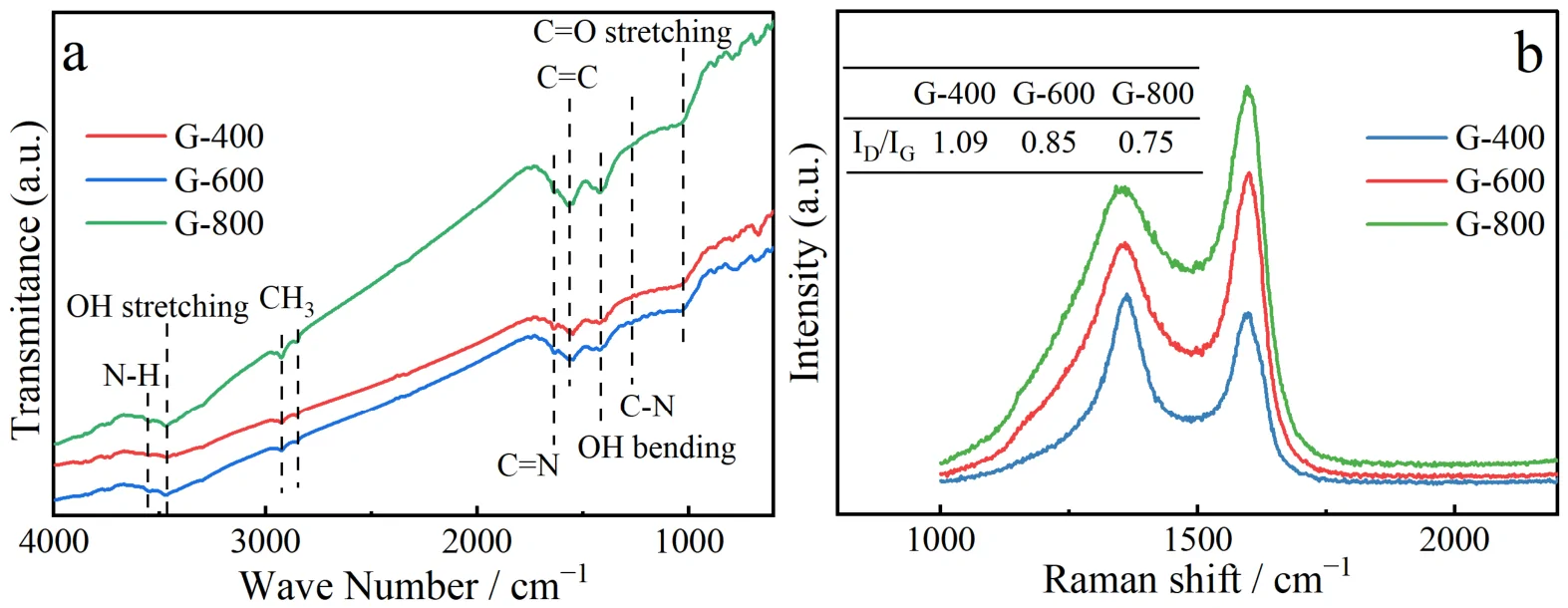
FT-IR (**a**) and Raman (**b**) spectra of N-doped graphene samples prepared at different temperatures.

The FT-IR spectra of all samples are shown in Figure 2a. A prominent characteristic band located at 1564 cm^−1^ corresponds to graphene, which originates from the skeletal stretching vibration of sp^2^ hybridized C=C bonds within the graphitic framework. Apart from this intrinsic graphene signal, multiple additional characteristic absorption signals were observed in the test samples. The broad absorption centered at 3467 cm^−1^ and its matched bending vibration mode at 1414 cm^−1^ are both assigned to hydroxyl (O-H) groups. The stretching vibration of carbonyl C=O gives rise to the band at 1025 cm^−1^. Two distinct bands at 2927 cm^−1^ and 2853 cm^−1^ stem from aliphatic C-H stretching vibrations [5,25]. Following thermal annealing under an NH_3_ atmosphere, a new stretching band at 3548 cm^−1^ is detected, which is attributed to N-H vibrations derived from secondary amine (-NH-) and primary amine (-NH_2_) moieties. A weak absorption signal at 1634 cm^−1^ is characteristic of C=N stretching modes. Furthermore, the band at 1273 cm^−1^ is ascribed to bending vibrations of aromatic C-N linkages [22].

Figure 2b shows the Raman spectra of the as-prepared samples. All spectra display the characteristic D and G bands of graphitic carbon, located at around 1360 cm^−1^ and 1600 cm^−1^, respectively. The D band originates from the breathing vibration of κ-point phonons possessing A_1_g symmetry. This phonon mode gains Raman activity once structural defects and disordered carbon domains break the fundamental Raman selection rules. In contrast, the G band corresponds to the doubly degenerate E_2_g phonon mode located at the Brillouin zone center, which derives from the in-plane stretching vibration of sp^2^-hybridized carbon atoms in the graphitic lattice. Consequently, the D band is associated with out-of-plane disordered carbon structures, while the G band characterizes the highly ordered in-plane sp^2^ graphitic network [26].

The intensity ratio of the D band to the G band (I_D_/I_G_) is a widely adopted metric to quantify structural disorder and graphitization level [27,28,29]. For each calcined sample, three independent Raman spectra were collected to obtain the uncertainty of the I_D_/I_G_ value. The calculated I_D_/I_G_ value of sample G-400 reached 1.09 ± 0.02. Upon elevating the NH_3_ annealing temperature, the I_D_/I_G_ value declined to 0.85 ± 0.01 and 0.75 ± 0.02 in sequence. This downward trend reflects defect healing and partial structural ordering during thermal treatment. Despite the gradual reduction in defect density, the relatively high I_D_/I_G_ values confirm that the material still possesses considerable intrinsic defects instead of forming fully ordered graphitic structures.

**Figure 3:**
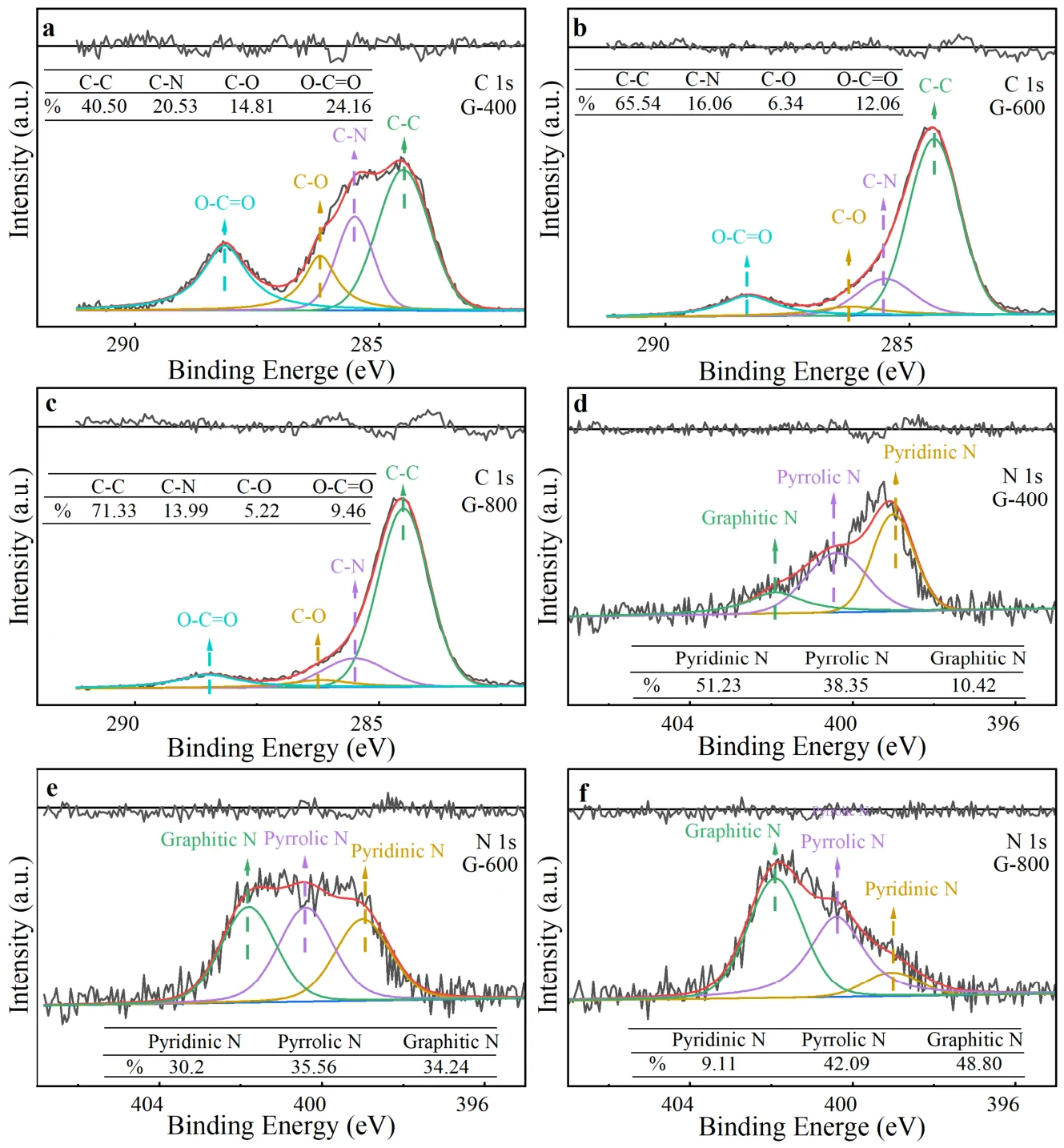
XPS spectra of N-doped graphene samples prepared at different temperatures. (**a**–**c**) present the C 1s core-level spectra of G-400, G-600, and G-800, respectively; (**d**–**f**) correspond to the N 1s core-level spectra of G-400, G-600, and G-800, respectively. Normalized residual curves for each peak fitting are displayed at the top of each subgraph.

XPS was used to characterize the elemental composition and chemical bonding states of N-doped graphene samples reacted under various annealing conditions. The spectra typically reveal characteristic peaks corresponding to the elemental composition, with distinct peaks for carbon and nitrogen. Specific functional groups on the surface are accountable for the binding energies described in the XPS spectra. The binding energy positions in high-resolution XPS spectra correspond to various surface functional groups [30,31,32,33]. All high-resolution C 1s spectra were calibrated using the sp^2^ C=C peak at 284.5 eV for charge correction. The peak deconvolution of the C 1s envelope identifies one dominant asymmetric component centered at 284.5 eV originating from sp^2^ hybridized carbon, alongside three minor subpeaks located at 285.5 eV (C-N bond), 286.3 eV (C-O bond), and 288.4–288.6 eV (O=C-O carboxylate groups) [24]. The relative content of each carbon component was quantitatively determined by calculating the integrated area ratio of each fitted peak. For the G-400 sample, the integrated area fraction of the sp^2^ carbon peak at 284.5 eV accounts for 40.5% of the total C 1s signal (see inset in Figure 3a), indicating that the continuous aromatic carbon network cannot be fully reconstructed after low-temperature NH_3_ annealing treatment. With the increase in NH_3_ annealing temperature, the sp^2^ carbon fraction of the G-600 and G-800 samples increases to 65.54% and 71.33%, respectively. This variation demonstrates that an elevated annealing temperature effectively promotes the reconstruction of continuous aromatic carbon networks in graphene. Within the entire annealing temperature range adopted in this work, the content of carbon–oxygen bonding configurations decreases significantly. Specifically, the relative contents of C-O bonds (286.2–286.3 eV) and O=C-O carboxylate groups (288.3–288.5 eV) decrease rapidly from 14.81% and 24.16% for the G-400 sample to 5.22% and 9.46% for the G-800 sample, respectively. Consistent with previous research results [34], the residual carbon–oxygen functional groups on the graphene surface are conducive to the preparation of high-quality graphene materials. In contrast, the content of C-N bonds exhibits negligible variation with annealing temperature. The relative contents of C-N bonds in the G-400, G-600, and G-800 samples are 16.53%, 16.03%, and 16.29%, respectively. Further analysis of high-resolution N 1s spectra confirms that the bonding configurations of nitrogen atoms undergo substantial changes during thermal annealing treatment.

The nitrogen bonding configurations of NH_3_ annealed samples were also systematically investigated via high-resolution N 1s XPS. The deconvolution of N 1s XPS spectra yielded three characteristic peaks, which correspond to different nitrogen doping configurations [35,36]. Specifically, the low binding energy peak at 398.8–398.9 eV is assigned to pyridinic N, the medium binding energy peak at 400.3–400.4 eV corresponds to pyrrolic N, and the high binding energy peak at 401.8–401.9 eV is attributed to graphitic N. The relative content of each nitrogen configuration was quantitatively calculated by the integrated area ratio of each fitted peak. The quantitative results, as presented in the insets of Figure 3d–f, reveal that annealing temperature exerts a significant modulation effect on the distribution of nitrogen configurations. For the low-temperature annealed G-400 sample, pyridinic N dominates the nitrogen configurations with a high proportion of 51.23%, while the graphitic N content is as low as 10.42%, indicating the low graphitization degree of the sample. With the increase in annealing temperature to 600 °C (G-600 sample), the proportion of pyridinic N decreases to 30.2%, whereas the content of graphitic N rises correspondingly to 34.24%. Further increasing the annealing temperature to 800 °C (G-800 sample) leads to a sharp elevation of graphitization degree, with the graphitic N content reaching 48.8% and the pyridinic N content dropping drastically to 9.11%. Notably, the pyrrolic N content remains relatively stable throughout the entire annealing temperature range, with values of 38.35%, 35.56%, and 42.09% for the G-400, G-600, and G-800 samples, respectively. The total nitrogen atomic concentration shows only minor differences among samples (0.41–0.46 at.%; see Table S1 and Figure S1 in Supporting Information). Therefore, the relative fractions of different N-configurations from N 1s deconvolution are adopted for the main-text discussion. Absolute atomic percentages of each nitrogen species are provided in Table S1 for reference.

Based on the above results, N-doped graphene was successfully fabricated via a facile two-step strategy combining solvothermal treatment and NH_3_ atmosphere annealing. In this synthetic route, metallic sodium reduced glucose to form graphene nanosheets, while subsequent NH_3_ annealing enabled effective nitrogen incorporation into the graphene lattice with tunable nitrogen configurations. The structural morphological, compositional, and defect characteristics of the obtained materials were systematically verified by SEM, XRD, FTIR, Raman, and XPS characterizations.

### 3.2. Room-Temperature Gas Sensing Properties of N-Doped Graphene

As widely documented in the previous literature [37], graphene holds great promise for the detection of various gaseous analytes including NO_2_ and CO. In this work, NH_3_ was selected as the target gas to systematically investigate the room-temperature gas sensing performance of N-doped graphene. As a typical industrial atmospheric pollutant, NH_3_ poses severe threats to human health even at trace concentrations at the ppm level. The gas sensor was fabricated via drop casting the as-synthesized graphene dispersion onto an alumina substrate preprinted with gold interdigitated electrodes. The sensing performance was characterized by monitoring the real-time conductance variations of the sensor upon exposure to target gas atmospheres with different concentrations.

**Figure 4:**
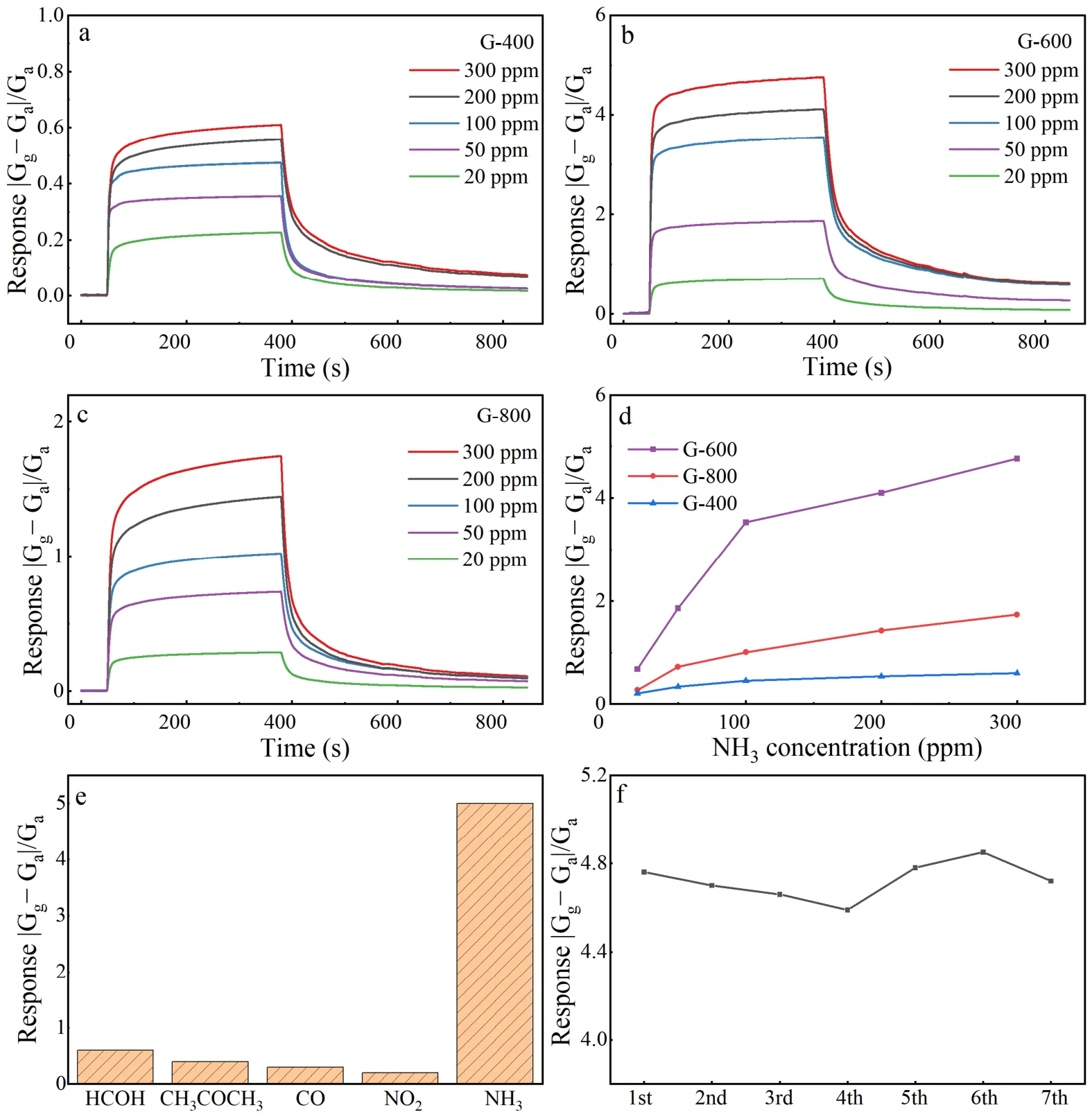
Dynamic response curves of N-doped graphene sensors toward NH_3_ gas with different concentrations: (**a**) G-400, (**b**) G-600, (**c**) G-800; (**d**) response values of N-doped graphene sensors at varying NH_3_ concentrations; (**e**) response values of the G-600 sensor toward different VOCs at 300 ppm; (**f**) cyclic response performance of the G-600 sensor toward 300 ppm NH_3_.

Benefiting from the regulated nitrogen doping configurations, the three samples exhibit distinct room-temperature gas sensing behaviors toward NH_3_. The dynamic sensing responses of the three sensors toward various NH_3_ concentrations at room temperature are displayed in Figure 4a–c, and the corresponding response–concentration correlation curves are summarized accordingly. Since NH_3_ serves as both the dopant source and reductive medium herein, a qualified undoped control sample under identical heat treatment conditions cannot be achieved. The performance differences between the three doped samples with varied nitrogen species therefore directly illustrate how nitrogen configurations modulate gas sensing properties. For the G-400 sensor, the sensing response increased from 0.22 to 0.61 when the NH_3_ concentration varied from 20 ppm to 300 ppm. Under the same test conditions, the response of the G-800 sensor increased from 0.28 to 1.74, while the G-600 sensor delivered a significantly enhanced response, rising from 0.69 to 4.76. Notably, the sensing response of the G-600 sensor toward 300 ppm NH_3_ is approximately 7.8 times higher than that of the G-400 sensor. These results demonstrate that the G-600 sensor possesses prominently improved room-temperature NH_3_ sensing sensitivity compared to its two counterparts.

Furthermore, the N-doped graphene sensor presents detectable sensing responses toward various volatile organic compounds (VOCs), including HCOH, CH_3_COCH_3_, CO, and NO_2_, as depicted in Figure 4e. Notably, the sensor delivers the maximum response amplitude toward NH_3_ among all tested gases, demonstrating the prominent selectivity of the N-doped graphene sensor for NH_3_ detection. Figure 4f displays the cyclic sensing performance of the optimized G-600 sensor toward 300 ppm NH_3_ over seven consecutive test cycles. The sensor maintains a stable response with negligible signal fluctuations throughout the cycling tests, confirming its outstanding reproducibility and operational stability. Collectively, these experimental results verify that the N-doped graphene sensors achieve superior room-temperature gas sensing performance, characterized by high sensitivity, favorable selectivity, and excellent stability. Compared with previously reported NH_3_ sensors [15,38,39,40,41,42], which are shown in Table 1, the comprehensive advantages of the as-fabricated sensors endow them with great application potential in indoor air pollution and industrial environment monitoring, particularly for safety detection in flammable and explosive environments.

### 3.3. Room-Temperature Gas Sensing Mechanism of N-Doped Graphene

Integrating the experimental results with the previously reported literature, this study systematically elucidates the underlying NH_3_ sensing mechanism of N-doped graphene. The N-doped graphene sensor fabricated herein is a typical p-type surface governed gas sensor, whose sensing performance is highly reliant on the surface adsorbed oxygen species. The positive Hall coefficients of all samples provide definitive experimental evidence for the p-type conduction of our N-doped graphene, as shown in Table S2. XPS characterizations verify that nitrogen doping configurations in graphene predominantly exist in three forms: pyridinic N, pyrrolic N, and graphitic N. In accordance with prior investigations [43], pyridinic N doping endows graphene with intrinsic p-type semiconductor characteristics, where holes dominate the carrier transport and abundant lattice vacancies are generated on the material surface. Under ambient atmospheric conditions, oxygen molecules spontaneously adsorb onto the surface of N-doped graphene sensing films. According to the Wolkenstein adsorption theory, neutral adsorbed oxygen species (O_2_)_ads_ capture valence band electrons and partially transform into reactive oxygen anions (including O^−^, O^2−^, and O_2_^−^). As our previous paper reported [6], under dry air and room-temperature conditions, molecular superoxide O_2_^−^ is the dominant adsorbed oxygen species on N-doped graphene, while atomic O^−^ or O^−2^ only forms at a high temperature (>100 °C) and can be neglected at room temperature. Such oxygen-induced electron depletion facilitates the formation of a hole accumulation layer and establishes a continuous negative charge layer across the sensor surface.

When exposed to a reductive NH_3_ atmosphere, surface adsorbed oxygen species react with NH_3_ molecules via a redox reaction. This process releases trapped electrons back to the valence band of N-doped graphene. The recovered electrons further recombine with holes within the accumulation layer, substantially reducing the carrier density and inducing a prominent variation in the resistance of the sensor. The exceptional NH_3_ sensing performance of N-doped graphene originates from the synergistic modulation of multiple N-doping configurations. Fundamentally, the NH_3_ sensing process follows a two-step pathway: the initial chemical adsorption of NH_3_ on the graphene surface and subsequent interfacial charge transfer between adsorbed NH_3_ species and the graphene substrate. The proposed fully balanced oxidation reaction equation for NH_3_ reacting with N-doped graphene can be described as follows:

O_2_ (gas) → O_2_ (ads) (1)

O_2_ (ads) + e^−^ → O_2_^−^ (ads) (2)

NH_3_ (gas) → NH_3_ (ads) (3)

4 NH_3_ (ads) + 5 O_2_^−^ (ads) → 4 NO (ads) + 6 H_2_O + 5 e^−^ (4)

where gas represents the substance in the gaseous state; ads represents the substance in the adsorbed state.

**Figure 5:**
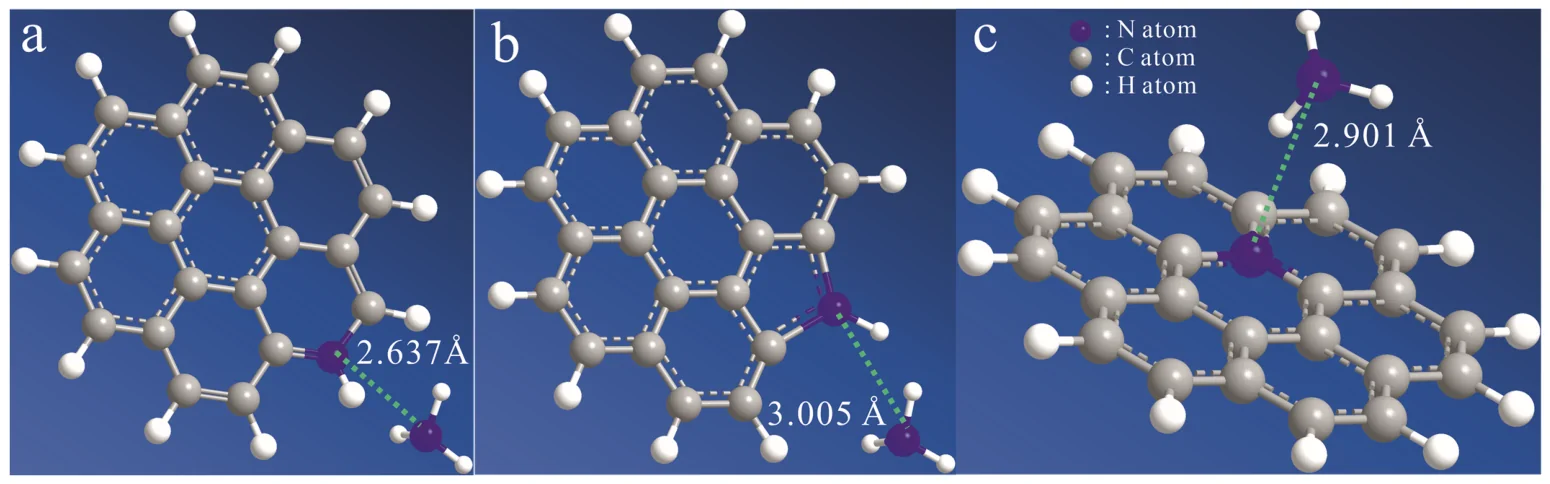
Optimized adsorption configurations of NH_3_ molecule on N-doped graphene with three typical nitrogen active sites: (**a**) pyridinic N, (**b**) pyrrolic N, and (**c**) graphitic N. The peripheral dangling bonds of graphene fragments are saturated with H atoms. Green dashed lines denote the intermolecular distances between NH_3_ and doped N sites. Blue sphere: N atom; gray sphere: C atom; white sphere: H atom.

DFT calculations were implemented to further uncover the intermolecular interaction mechanism between the N-doped graphene and NH_3_ molecules. Corresponding to the three N configurations identified by XPS, independent adsorption models were constructed for pyridinic N, pyrrolic N, and graphitic N, with the key computational results summarized in Table 2. The structure-optimized adsorption configurations of NH_3_ on three typical N-doped graphene models are displayed in Figure 5. The calculated adsorption energies of NH_3_ on pyridinic N, pyrrolic N, and graphitic N are −0.26 eV, −0.08 eV, and −0.12 eV, respectively. Simulation results confirm that pyridinic N possesses the strongest NH_3_ adsorption capability and the most pronounced interfacial charge transfer behavior. Specifically, the Mulliken population analysis reveals a net electron density transfer of 0.04 e from NH_3_ to the pyridinic N graphene substrate. This fractional charge redistribution modulates the Fermi level and carrier concentration of the substrate. Meanwhile, the optimized adsorption configuration delivers a minimum intermolecular distance of 2.637 Å between NH_3_ and the pyridinic N plane, verifying the formation of enhanced adsorption. Such interfacial behavior corresponds to charge-transfer-assisted adsorption. From the perspective of Lewis acid/base chemistry, NH_3_ serves as a Lewis-base electron donor with lone-pair electrons. Pyridinic-N doping induces local lattice polarization and produces electron-deficient carbon sites in its vicinity, which act as Lewis-acid sites to interact with NH_3_. Accordingly, theoretical simulations demonstrate that pyridinic N exhibits the highest affinity toward NH_3_ among all doping configurations.

Structurally, pyridinic N contains lone-pair electrons that enable synergistic hydrogen bonding and electrostatic interactions with hydrogen atoms in NH_3_ molecules, thereby generating much stronger chemical adsorption compared with graphitic N and pyrrolic N. As a typical edge-doping motif, pyridinic N introduces hole carriers into the graphene framework and realizes effective p-type modulation. As an electron-donating reductive gas, adsorbed NH_3_ neutralizes the accumulated holes, triggering significant resistance variation and thus enhancing the sensing response. Furthermore, the intrinsic edge defect features of pyridinic N endow the material with superior adsorption selectivity toward alkaline NH_3_ over common interfering gases, including acetone, CO, and formaldehyde, thereby guaranteeing the excellent anti-interference capability of the sensor.

In sharp contrast, pyrrolic N presents negligible NH_3_ adsorption activity, reflected by a low adsorption energy of −0.08 eV, a long adsorption distance of 3.005 Å, and a minimal charge transfer of 0.01 e. The weak intermolecular interaction between pyrrolic N and NH_3_ is dominated by van der Waals forces, without stable hydrogen bond formation. Consequently, pyrrolic N cannot function as an effective sensing active site, and excessive pyrrolic N incorporation inevitably compromises the overall sensing performance.

Unlike pyridinic N and pyrrolic N, graphitic N acts as an in-plane substitutional dopant that incorporates abundant free electrons into the conjugated graphene framework. Such electronic modulation effectively not only rearranges the surface electron distribution of graphene and elevates the Fermi level, modulating the strength of Lewis acid/base interaction between NH_3_ (Lewis-base electron donor) and electron-deficient polarized carbon sites adjacent to nitrogen dopants [44], but also reduces the initial resistance of the sensing film and facilitates carrier migration [45]. Distinct from edge-located pyridinic N that induces substantial lattice defects and strong gas adsorption yet sluggish molecular desorption, lattice-embedded graphitic N maintains the integrity of the sp^2^ carbon lattice and suppresses carrier scattering. The superior electrical conductivity and efficient charge transport capability enabled by graphitic N can favorably compensate for the slow desorption drawback originating from the strong adsorption affinity of pyridinic N, achieving a balanced sensing performance. This conductivity regulation mechanism of graphitic N has been experimentally validated [46], demonstrating that in-plane graphitic N doping is conducive to constructing continuous and high-efficiency conductive networks in graphene-based sensing materials. Although single-N-species samples cannot be obtained, the electrical properties of G-400, G-600, and G-800 samples were measured by the sheet resistance and Hall mobility via the four-probe method, as shown in Figure S2. The measured sheet resistance values are 28.5 ± 2.3 kΩ/sq, 26.3 ± 2.1 kΩ/sq, and 18.5 ± 1.2 kΩ/sq for G-400, G-600, and G-800 samples, respectively. As a direct consequence of the decrease in the sheet resistance, it was observed that the Hall mobility of the charge carriers increases from 10.4 ± 2.4 to 36.4 ± 3.1 cm^2^ V^−1^ s^−1^ with an increase in the annealing temperature. In this case, the Hall mobility mainly increases due to the high orders and recovery of the continuity in the sp^2^ carbon network and suppresses carrier scattering in the graphene lattice, thereby improving the electrical conductivity of the N-doped graphene. As shown in the XPS results, with the increase in calcination temperature, the relative content of graphitic N rises, while pyridinic N and pyrrolic N decrease synchronously. Correspondingly, the sheet resistance gradually decreases, demonstrating improved electrical conductivity. Also, the sign of the measured Hall coefficient is positive for all N-doped graphene samples in this work, which unambiguously demonstrates that holes act as majority charge carriers (intrinsic p-type conduction). Positive R_H_ values would indicate hole-dominated p-type transport, whereas negative R_H_ values would confirm an electron conduction channel.

The comprehensive NH_3_ sensing performance of N-doped graphene is governed by the synergistic balance between pyridinic N-dominated adsorption activity and graphitic N-modulated electrical conductivity, which fundamentally explains the optimal sensing behavior of the G-600 sample. Consistent with previous DFT calculations and experimental findings, pyridinic N serves as the primary active center for NH_3_ detection. The unique edge defects and unsaturated dangling bonds introduced by pyridinic N significantly promote the enhanced adsorption of reductive NH_3_ molecules and improve interfacial charge transfer efficiency. In comparison, in-plane substituted graphitic N maintains sp^2^ lattice integrity, suppresses carrier scattering, and provides additional free electrons, thereby effectively boosting the electrical conductivity and charge transport efficiency of graphene-based sensors.

For the G-400 sample, the insufficient graphitic N content limits the electrical conductivity of the material. Although the abundant pyridinic N guarantees a favorable NH_3_ adsorption capacity, the poor charge migration efficiency restricts the effective transmission of sensing signals, resulting in a relatively low response sensitivity. In comparison, the excessive doping degree of the G-800 sample significantly increases the proportions of graphitic N and pyrrolic N. Despite the excellent electrical conductivity of G-800 enabled by the high graphitic N concentration, the sharp reduction in pyridinic N active sites severely weakens the NH_3_ chemical adsorption capacity and interfacial charge transfer behavior, leading to insufficient gas adsorption activity and degraded sensing performance.

Benefiting from the synergistic optimization of dual nitrogen doping configurations, the G-600 sample achieves an ideal trade-off between adsorption activity and electrical conductivity. The moderate pyridinic N content provides sufficient active sites for stable NH_3_ adsorption and efficient charge injection, while the appropriate graphitic N component guarantees superior carrier mobility and continuous conductive network transmission. A prominent synergistic coupling effect exists between pyridinic N and graphitic N in optimizing NH_3_ sensing performance. Specifically, high-mobility graphitic N constructs a high-efficiency conductive network that effectively collects and transmits electrons transferred from adsorbed NH_3_ as valid sensing signals. By contrast, pyridinic N acts as the core reaction center with strong NH_3_ affinity and highly efficient interfacial charge interaction. The integration of its high adsorption activity from pyridinic N and its excellent charge transport capability from graphitic N endows the G-600 sample with an optimal comprehensive NH_3_ sensing performance.

## 4. Conclusions

In summary, N-doped graphene with controllable nitrogen configurations was successfully prepared by a two-step method combining solvothermal reduction and NH_3_ annealing at varied temperatures. The structural and compositional analyses confirmed that increasing annealing temperature progressively converts pyridinic N to graphitic N while pyrrolic N remains relatively stable. Room-temperature NH_3_ sensing measurements demonstrated that the G-600 sensor achieves the best overall performance, with a response of 4.76 at 300 ppm, favorable selectivity toward NH_3_ against the four typical interfering gases (HCHO, CO, CH_3_COCH_3_, and NO_2_), and excellent cyclic reproducibility. DFT calculations revealed that pyridinic N acts as the primary enhanced adsorption site due to its lone-pair electrons and edge defect nature, enabling strong NH_3_ adsorption and efficient interfacial charge transfer, while graphitic N facilitates carrier mobility and conductive network integrity. Therefore, the synergistic cooperation between pyridinic N and graphitic N is the key to optimizing the sensing performance. This study provides a clear atomic-level understanding of the role of different nitrogen configurations and establishes a design guideline for fabricating high-efficiency, room-temperature NH_3_ gas sensors based on N-doped graphene, with great potential for practical applications in industrial safety, environmental monitoring, and hydrogen energy systems.

## Figures and Tables

**Table 1:** Comparison of sensing capabilities in this work with those of NH_3_ sensors reported in the literature at room temperature.

Sensing Material	Concentration (ppm)	Response	Reference
N-doped graphene	300	4.76	this work
ZnO/GO	50	0.8	[38]
CuO/rGO	50	0.18	[15]
N-doped rGO/ZnO	200	0.18	[39]
ZnO/GO	50	0.6	[40]
rGO	25	0.098	[41]
PANI/SrGeO_9_	20	0.16	[42]

GO: graphene oxide, rGO: reduced graphene oxide, PANI: polyaniline.

**Table 2:** DFT calculation results of the adsorption energy (*E*_a_), Mulliken charge (*Q*), and shortest atom-to-atom distance (*d*) for NH_3_ molecule adsorption on the N-doped graphene model molecule.

System	E_a_ (eV)	Q^a^ (e)	D (Angstrom)
pyridinic N	−0.26	0.04	2.637
pyrrolic N	−0.08	0.01	3.005
graphitic N	−0.12	0.02	2.901

E_a_ is the adsorption energy, E_a_ = E_N-doped graphene + NH3_ − E_N-doped graphene_ − E_NH3_; d is the average distance; Q is the Mulliken charge on the gas molecule. ^a^ A positive number indicates an electron transfer from gas molecules to N-doped graphene, while negative numbers indicate an electron transfer from N-doped graphene to NH_3_ gas molecules.

## Data Availability

All data generated in this study are available within the paper.

## Supplementary Materials

The following supporting information can be downloaded at: https://www.mdpi.com/article/10.3390/nano16161044/s1, Figure S1: Survey XPS full-scan spectra of G-400, G-600 and G-800 samples. The inset table summarizes the atomic percentages (at%) of C, O and N derived from survey-spectrum quantitative analysis; Figure S2: Sheet resistance and and Hall mobility of G-400, G-600 and G-800 samples; Table S1: Total nitrogen atomic fraction and calculated absolute atomic percentages of pyridinic-N, pyrrolic-N, and graphitic-N for G-400, G-600 and G-800. Table S2: Electrical transport data from Hall measurements for N-doped graphene.

## Abbreviations

The following abbreviations are used in this manuscript:

CCD	Charge-coupled device
MOS	Metal oxide semiconductor
ACGIH	American Conference of Governmental Industrial Hygienists
XPS	X-ray photoelectron spectroscopy
FE-SEM	Field-emission scanning electron microscopy
XRD	X-ray diffraction
DFT	Density functional theory
FTIR	Fourier transform infrared spectroscopy
ppm	Parts per million
BSSE	Basis-set superposition error
VOC	Volatile organic compound
