# Supplementary material for: Nitrogen-Doped Graphene with Enhanced Room Temperature NH3 Gas Sensing Properties
Source: Nanomaterials (Basel). 2026 Aug 21;16(16):1044. doi: 10.3390/nano16161044 (PMC13516361; doi:10.3390/nano16161044)
Supplement: Supplementary file 1 [file nanomaterials-16-01044-s001.zip › nanomaterials-4477982-supplementary.pdf]

Supplementary Materials

Nitrogen-Doped Graphene with Enhanced Room Temperature NH_3 Gas Sensing Properties

Qingwu Huang [1], Peng Zhou [1], Wulin Song [1,2] and Jinjin Wu [1,] *

[1] Analytical and Testing Center of Huazhong University of Science and Technology, Huazhong University of Science and Technology, No. 1037, Luoyu Road, Wuhan 430074, China; qwhuang@hust.edu.cn (Q.H.); zhoupeng_peng@hust.edu.cn (P.Z.); wulins@126.com (W.S.)

[2] State Key Laboratory of Materials Processing and Die & Mould Technology, Huazhong University of Science and Technology, No. 1037, Luoyu Road, Wuhan 430074, China

* Correspondence: jjwu@hust.edu.cn; Tel.: +86-027-87793949

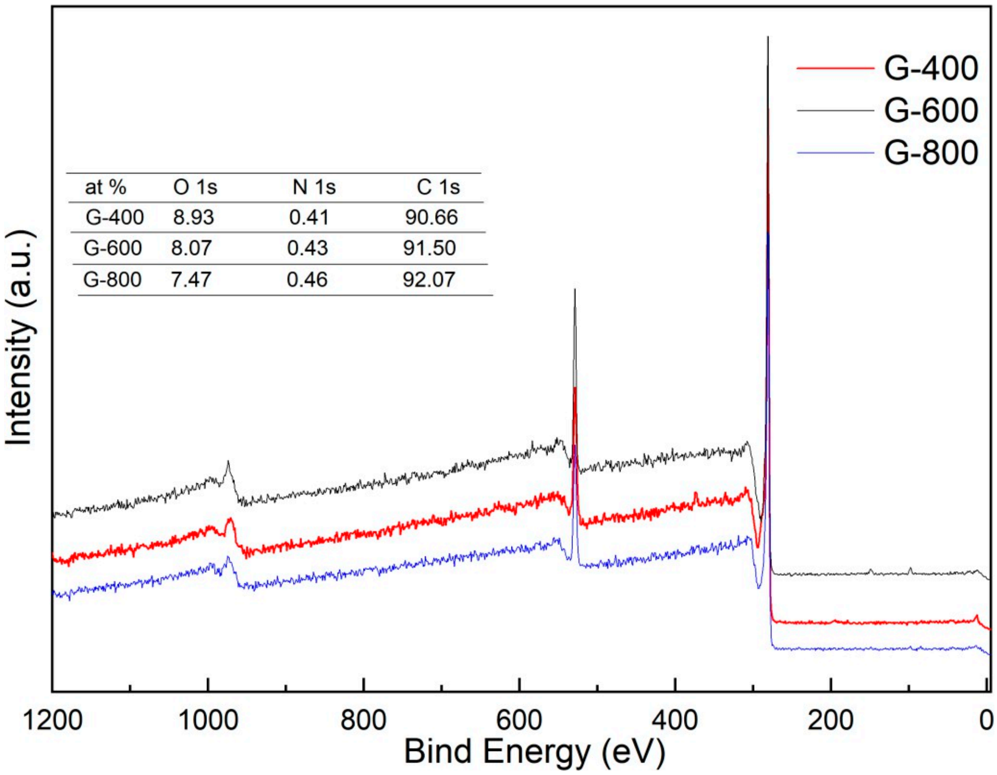

Figure S1. Survey XPS full-scan spectra of G-400, G-600 and G-800 samples. The inset table summarizes the atomic percentages (at%) of C, O and N derived from survey-spectrum quantitative analysis.

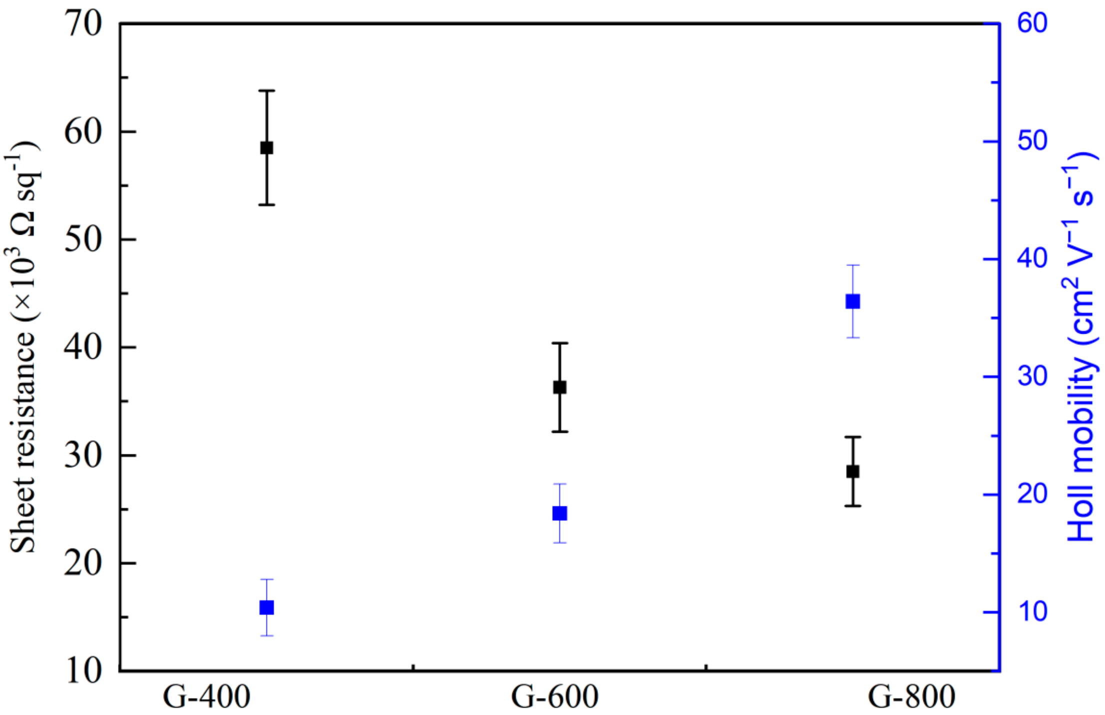

Figure S2. Sheet resistance and and Hall mobility of G-400, G-600 and G-800 samples.

Error bars represent the standard deviation from three parallel measurements.

Table S1. Total nitrogen atomic fraction and calculated absolute atomic percentages of pyridinic-N, pyrrolic-N, and graphitic-N for G-400, G-600 and G-800.

Sample	Total N (at. %)	Pyridinic-N (at. %)	Pyrrolic-N (at. %)	Graphitic-N (at. %)
G-400	0.41	0.21	0.16	0.04
G-600	0.43	0.13	0.15	0.15
G-800	0.46	0.04	0.19	0.22

Absolute atomic percentages of each N species are calculated by multiplying the total N atomic concentration from survey XPS by the relative fractions of N 1s deconvolution.

Table S2. Electrical transport data from Hall measurements for N-doped graphene.

Sample	Sheet resistance ($k\Omega \cdot sq^{-1}$)	Hall mobility ($cm^2 \cdot V^{-1} \cdot s^{-1}$)	Hall coefficient R_H ($cm^3 \cdot C^{-1}$)	Carrier type
G-400	58.5 ± 5.3	10.4 ± 2.4	+7374 ± 2692	p-type
G-600	36.3 ± 4.1	18.4 ± 2.5	+6900 ± 1997	p-type
G-800	28.5 ± 3.2	36.4 ± 3.1	+8320 ± 2182	p-type
